# Cytoprotective Mechanisms Mediated by Polyphenols from Chilean Native Berries against Free Radical-Induced Damage on AGS Cells

**DOI:** 10.1155/2017/9808520

**Published:** 2017-05-02

**Authors:** Felipe Ávila, Cristina Theoduloz, Camilo López-Alarcón, Eva Dorta, Guillermo Schmeda-Hirschmann

**Affiliations:** ^1^Escuela de Nutrición y Dietética, Facultad de Ciencias de la Salud, Universidad de Talca, 3460000 Talca, Chile; ^2^Laboratorio de Cultivo Celular, Facultad de Ciencias de la Salud, Universidad de Talca, 3460000 Talca, Chile; ^3^Departamento de Química Física, Facultad de Química, Pontificia Universidad Católica de Chile, 7820436 Santiago, Chile; ^4^Laboratorio de Química de Productos Naturales, Instituto de Química de Recursos Naturales, Universidad de Talca, 3460000 Talca, Chile

## Abstract

The prevalence of cytoprotective mechanisms induced by polyphenols such as activation of intracellular antioxidant responses (ICM) and direct free radical scavenging was investigated in native Chilean species of strawberries, raspberries, and currants. Human gastric epithelial cells were co- and preincubated with polyphenolic-enriched extracts (PEEs) from Chilean raspberries (*Rubus geoides*), strawberries (*Fragaria chiloensis* ssp. *chiloensis* f*. chiloensis*), and currants (*Ribes magellanicum*) and challenged with peroxyl and hydroxyl radicals. Cellular protection was determined in terms of cell viability, glyoxalase I and glutathione s-transferases activities, and carboxymethyl lysine (CML) and malondialdehyde levels. Our results indicate that cytoprotection induced by ICM was the prevalent mechanism for *Rubus geoides* and *F. chiloensis*. This agreed with increased levels of glyoxalase I and glutathione S-transferase activities in cells preincubated with PEEs. ORAC index indicated that *F. chiloensis* was the most efficient peroxyl radical scavenger. Moreover, ICM mediated by *F. chiloensis* was effective in protecting cells from CML accumulation in contrast to the protective effects induced by free radical scavenging. Our results indicate that although both polyphenol-mediated mechanisms can exert protective effects, ICM was the most prevalent in AGS cells. These results suggest a potential use of these native berries as functional food.

## 1. Introduction

Oxidative stress has been implied in the etiology of numerous diseases as well as in the ageing process [1–3]. The balance established in terms of the relative amounts of antioxidants and reactive species (reactive oxygen and nitrogen species and electrophilic molecules) plays a pivotal role in the cellular integrity and functionality [4]. Antioxidants and reactive species can have an exogenous or endogenous origin, dietary intake being the most important source of exogenous antioxidants [5]. It has been suggested that the intake of dietary antioxidants is inversely associated with the development of chronic diseases, which currently constitute the main cause of mortality worldwide [6]. In fact, dietary interventions in humans, as well as in vitro studies, have shown evidence regarding the beneficial effects on the health related to the consumption of berries [7]. These effects have been mainly attributed to their content of polyphenols, which can protect biological systems against the damage induced by numerous agents, including free radicals [8, 9]. Cellular protection mediated by polyphenols against free radical-induced damage can be exerted by different mechanisms, including scavenging of free radicals by direct reactions, metal ion complexation with a consequent inhibition of the free radicals generated through Fenton-type reactions, and activation of the intracellular signaling cascades that result in activation of detoxifying enzymes or repression of pro-oxidant proteins [10–12]. In addition, during the last years, it has been suggested that, under physiological conditions and in vivo, the main protective mechanism associated with polyphenols arising from dietary sources involves activation of transcription factors that regulate the expression and activity of antioxidant and detoxifying systems in cells [12]. This concept agrees with the relative low bioavailability of polyphenols (implying very low systemic concentrations) and would explain the lack of correlation between their in vitro antioxidant capacity and their protective effects on cellular cultures (which imply cellular responses) [10].

On the other hand, human gastric epithelial cells are constantly exposed to an oxidative environment produced by the intake of reactive species (generated during thermal processing of foods) as well as by the oxidative reactions that take place during the processes associated with food digestion [13]. These cells are also exposed to polyphenols arising from foods, which have not been previously metabolized by the liver or colonic microflora [14]. In this sense, gastric epithelial cells constitute a unique model in which different mechanisms of cytoprotection mediated by polyphenols could be active. The human gastric adenocarcinoma cell line (AGS) consists of mucus-secreting epithelial cells presenting several characteristics of normal gastric epithelial cells and a good power of differentiation [15]. For this reason, they have been widely used to evaluate the antioxidant activity of several lentil cultivars [9, 16]. In fact, we have recently reported that polyphenolic-enriched extracts (PEEs) from Chilean native berries, including *Rubus geoides* and *Ribes magellanicum* (Figure S1 in Supplementary Material available online at https://doi.org/10.1155/2017/9808520), present a significant cytoprotective activity against H_2_O_2_ and methylglyoxal-induced damage of AGS cells [16]. The Chilean native berries selected for this study are wild relatives of the widely cultivated and consumed strawberries, raspberries, and currants. The PEEs of these species have also shown a high scavenging capacity of stable free radicals such as DPPH and ABTS [9, 16]. However, evidence aiming to elucidate the main mechanisms involved in the protection of gastric human cells mediated by polyphenols against the damage inflicted by free radicals is lacking.

It has been reported that numerous polyphenols are able to activate transcription factors including the nuclear factor-erythroid 2 p45 (Nrf2) [17, 18], which is involved in the control of the expression of detoxifying enzymes such as glutathione S-transferases [19] and glyoxalase I [20], inducing protective effects against oxidative stress [21]. Cellular activation of Nrf2 and the subsequent expression of detoxifying enzymes have shown to occur in a period of hours, and consequently, most responses associated to intracellular antioxidant response activation involves 16–24 hours of incubations [9, 22]. Such time frame contrasts with the kinetic of reactions between free radicals and polyphenols which typically involves rate constants in the range of 10^9^ M^−1^ s^−1^ (towards hydroxyl radicals) [11] to 10^3^ M^−1^ s^−1^ (towards superoxide radical anion) [23].

To get more insights regarding the protection afforded by polyphenols from the South American relatives of commercial berries on AGS cells exposed to a free radical source, in the present work, we have addressed studies aimed to assess the cytoprotective mechanisms mediated by the polyphenols contained in the Chilean native raspberry *Rubus geoides*, the native currant *Ribes magellanicum*, and the Chilean strawberry *Fragaria chiloensis* ssp. *chiloensis* f*. chiloensis*.

## 2. Materials and Methods

### 2.1. Chemicals

2,2′-Azobis(2-methyl-propionamidine) dihydrochloride (AAPH), copper sulphate, Chelex® 100 sodium, quercetin, thiobarbituric acid, trichloroacetic acid, Amberlite XAD-7, sodium bicarbonate, methylglyoxal, glutathione (reduced form), 1-chloro-2,4-dinitrobenzene (CDNB), L-glutamine, and CelLytic™ M were purchased from Sigma-Aldrich (St. Louis, MO, USA). Pierce™ BCA Protein Assay Kit was obtained from Thermo Scientific (Rockford, IL, USA). Hydrogen peroxide (30%), HCl, methanol, and formic acid were purchased from Merck (Darmstadt, Germany). MTT was obtained from Calbiochem (Darmstadt, Germany). Fetal bovine serum (FBS), antibiotics, and culture media were purchased from Invitrogen (Grand Island, New York, USA). Ultrapure water was obtained using a Barnstead EasyPure water filter system (Thermo Scientific, Dubuque, IA, USA).

### 2.2. Polyphenolic-Enriched Extracts (PEEs)

The Chilean berries included two collections from the native raspberry *Rubus geoides*, one of them from southern Chile (Las Raices) and the second one from Tierra del Fuego, Patagonia (Lago Blanco), the native currant *Ribes magellanicum* from Araucanía, southern Chile, and the Chilean strawberry *Fragaria chiloensis* ssp. *chiloensis* f*. chiloensis* was cultivated in central Chile (Figure S1). The study was performed with the polyphenolic-enriched extracts (PEEs) of the fruits. The PEEs were obtained according to the methodology previously reported [9, 24–26]. Briefly, the fruits were homogenized and extracted with a MeOH : formic acid 99 : 1 (*v*/*v*) mixture and the solution was taken to dryness under reduced pressure. The extract was resuspended in water and loaded on a preconditioned Amberlite XAD-7 resin. The resin was washed with distilled water, and the polyphenols were desorbed with MeOH to afford after concentration under reduced pressure and lyophilization of the phenolic-enriched extracts (PEEs). The chemical characterization of the PEEs has been reported as follows: *Ribes magellanicum* [16, 24], *Rubus geoides* [9], and *Fragaria chiloensis* ssp. *chiloensis* f*. chiloensis* [25, 26].

### 2.3. AGS Cell Culture

Human epithelial gastric cells (AGS) (ATCC CRL-1739) were grown as monolayers in HAM F-12 medium containing 1 mM L-glutamine and 1.5 g/L sodium bicarbonate, supplemented with 10% FBS, 100 IU/mL penicillin, and 100 *μ*g/mL streptomycin. Cells were grown in a humidified incubator with 5% CO_2_ in air at 37°C. For the subsequent experiments, cells were plated at a density of 2.5 × 10^4^ cells/mL.

### 2.4. Cell Viability Determination

Cell viability was determined by means of the MTT reduction assay [15]. Cytoprotection against free radical-induced damage was assessed by coincubation and preincubation models, according to the procedure described next. All the analyses were performed according to the following common procedures: untreated cells were used as 100% viability controls. Cells coincubated or preincubated with quercetin (6 *μ*g/mL) were used as positive controls [27]. Cells treated with the free radicals served as damage controls. Each concentration was tested in quintuplicate, and experiments were repeated two times using different cell preparations. Results are expressed as percentage of the 100% viability control.

#### 2.4.1. Coincubation Model (Free Radical Scavenging)

Confluent cultures of AGS cells were coincubated during 30 min or 60 min with the free radicals (see Section 2.5) together with different final concentrations of the PEEs as follows. For *R. geoides* and *R. magellanicum*, the concentrations are as follows: 0, 15.6, 31.3, 62.5, and 125 *μ*g/mL; for *F. chiloensis*: 0, 7.8, 15.6, 31.3, and 62.5 *μ*g/mL. The PEEs and the free radical sources were prepared freshly in a medium without antibiotics or FBS. At the end of the stress induction, the medium was removed by vacuum aspiration and replaced by complete medium (containing 10% of FBS). Cells were left to recover for 24 hours and cell viability was determined.

#### 2.4.2. Preincubation Model (Intracellular Antioxidant Responses)

Confluent cultures of AGS cells were preincubated during 16 h with different concentrations of the PEEs as described in Section 2.4.1. The extracts were dissolved in complete medium supplemented with 2% FBS. At the end of the incubation, culture medium was completely removed by vacuum aspiration. Then, the cells were submitted to the free radical-induced stress (see Section 2.5) using the radical sources freshly prepared in a medium without antibiotics or FBS. After the stress induction, the medium was removed by vacuum aspiration and replaced by complete medium (containing 10% of FBS). Cells were left to recover for 24 hours and cell viability was determined.

#### 2.4.3. Challenge with Hydroxyl Radical

Hydroxyl radicals were generated through a Fenton-type reaction [28]. Cell culture medium (HAM-F12), without FBS or antibiotics, was pretreated with Chelex to decrease the concentration of trace metals that could be present in the media. For cell viability analyses, the reaction was generated with different concentrations of hydrogen peroxide (0.5, 1, 1.5, and 2 mM) and different concentrations of CuSO_4_ (ranging from 0.2 *μ*M to 410 *μ*M). Controls incubated only with CuSO_4_ or hydrogen peroxide were also performed. Confluent cells were co- or preincubated with the different PEEs during 30 minutes at 37°C. Cells were left to recover incubating with complete medium (containing 10% of FBS) during 24 hours, and the effects were determined by means of MTT analysis according to the procedure described in Section 2.4.

#### 2.4.4. Peroxyl Radicals

Peroxyl radicals were generated by the thermal decomposition of 2,2′-azobis(2-methylpropionamidine) dihydrochloride (AAPH) [29]. Cell culture medium (HAM-F12), without FBS or antibiotics, was pretreated with Chelex. The cytotoxicity elicited by peroxyl radicals was evaluated, incubating during 60 minutes confluent AGS cells with different concentrations of AAPH (0.78 to 400 mM). Then, the solution was removed and cells were left to recover during 24 hours with FBS at 10%. Cell viability analyses were performed according to the procedure described in Section 2.4.

### 2.5. Enzymatic Activity Determination

AGS cells were seeded in 75 cm^2^ culture flasks until confluence. Then, cells were co- or preincubated (see Sections 2.4.1 and 2.4.2) with the PPEs from the selected species as follows: *R. geoides* (Las Raices) 62.5 *μ*g/mL, *R. geoides* (Lago Blanco) 125 *μ*g/mL, *R. magellanicum* 31.3 *μ*g/mL, and *F. chiloensis* 62.5 *μ*g/mL. At the end of the experiments, cells were collected using a cell scraper. Cells were centrifuged at 3500 rpm during 10 minutes to remove the culture medium. The pellet was kept at −80°C until analyses. On the day of the corresponding analysis, cells were lysed by adding 100 *μ*L of CelLytic and debris was removed by centrifugation at 10,000 rpm during 10 minutes. Protein concentration was determined by means of the bicinchoninic acid (BCA) method [30].

#### 2.5.1. Glutathione S-Transferases

The enzymatic activity of glutathione S-transferases (GST) was quantified according to the method of Habig and Jakoby [31]. The reaction mixture of 1 mL included 1 mM of 1-chloro-2,4-dinitrobenzene (CDNB), 1 mM of reduced glutathione, and 15 *μ*g of protein from treated cell extracts in 100 mM phosphate buffer pH 6.5. The reaction was initiated by the addition of the cell extract, and it was monitored by following the rate of the formation of the adduct glutathione-DNB (GS-DNB) at 340 nm. The initial rates were obtained from the slopes of plots of GS-DNB concentration (using an extinction coefficient of 0.0096 *μ*M^−1^ cm^−1^ at 340 nm) against time. The GST activity was expressed as a percentage, considering 100% as the slope of the controls (cells treated with medium only).

#### 2.5.2. Glyoxalase I (GLOI)

The enzymatic activity of glyoxalase I was determined spectrophotometrically, according to the protocol of Thornalley, with minor modifications [32]. Methylglyoxal (2 mM) was incubated during 5 minutes with reduced glutathione (2 mM) at pH 6.6 and 25 ± 2°C to produce a hemithioacetal by a nonenzymatic reaction. Then, 30 *μ*g of cellular protein extract was added and the increase in the absorbance due to S-D-lactoylglutathione formation was registered at 240 nm (*ε*_240_ = 2.86 mM^−1^ cm^−1^). The activities were assessed determining the initial rates of the S-D-lactoylglutathione formation. The GLOI activity was expressed as a percentage, considering 100% as the slope of the controls (cells treated with medium only).

### 2.6. Oxygen Radical Absorbance Capacity (ORAC)

Fluorescein- and pyrogallol red-based ORAC assays (ORAC-FL and ORAC-PGR) were determined according to Cao et al. [29] and Lopez-Alarcón and Lissi [33], respectively. A reaction mixture containing fluorescein (FL, 70 nM final concentration) or pyrogallol red (PGR, 5 *μ*M final concentration) was prepared in phosphate buffer (75 mM, pH 7.4), with or without the PEEs (50 *μ*g/mL, final concentration). The fluorescence (FL, *λ*_em_ = 515 nm; *λ*_ex_ = 493 nm) or the absorbance (PGR, *λ* = 540 nm) decay was measured using a multimode microplate reader (Synergy HTX; Biotek Instruments, Winooski, VT, USA). Stock solutions of the PEEs were prepared in ethanol at 5 mg/mL. The solutions were preincubated for 30 min at 37°C. The AAPH solution (10 mM final concentration) was added and the fluorescence (F) or absorbance (A) were registered every 30 s for 180 min. Data were plotted as *F*/*F*_0_ or *A*/*A*_0_ as a function of time. The area under the curve (AUC) of *F*/*F*_0_ or *A*/*A*_0_ was calculated. Plots and integration data were obtained using MicroCal Origin® 7.0 software (Boston, MA, USA). AUC data were used to obtain ORAC values, according to the equation described next. All experiments were carried out in triplicate.

(1) $$\begin{array}{c} ORAC=\frac{\left[AUC-{AUC}^{0}\right]}{\left[{AUC}_{Trolox}-{AUC}^{0}\right]}f\left[Trolox\right], \end{array}$$

where AUC corresponds to the experiments performed in the presence of the tested samples, integrating between time zero and the time corresponding to 80% of FL or PGR consumption; AUC^0^ corresponds to control experiments (in the absence of samples); AUC_Trolox_ corresponds to experiments in the presence of Trolox; *f* is the dilution factor (ratio between the final volume of the AAPH-FL or AAPH-PGR solutions and the added sample volume); and [Trolox] is the Trolox concentration (mM).

### 2.7. Thiobarbituric Acid Reactive Species (TBAR) Determination

The TBAR determination was carried out according to [34]. Briefly, a mixture of 20% (*w*/*v*) trichloroacetic acid and 0.8% (*w*/*v*) thiobarbituric acid was prepared in 0.25 N HCl (color reagent). The PEE from *F. chiloensis* (62.5 *μ*g/mL) was selected for this assay. Cells were grown until confluence in 5 cm diameter Petri dishes (19.6 cm^2^), treated under both schemes of co- and preincubations with the PEE, and challenged with AAPH 168 mM during 1 h. At the end of the incubation, the medium was removed by vacuum aspiration. One mL of the color reagent was added and cells were scrapped-off immediately and homogenized. Samples were quantitatively transferred into a glass vial and hermetically sealed with a vial crimper (Chromatography Research Supplies, USA) and then boiled during 45 min. Samples were allowed to cool-down to room temperature and centrifuged at 10,000 rpm for 10 min. The absorbance of the supernatant was measured at 535 nm, and the MDA concentration was calculated using the molar extinction coefficient of the MDA-TBA_2_ complex of 1.49 × 10^5^ M^−1^ cm^−1^. The results were expressed as nmol of MDA/mg of protein. Additional control plates with AGS cells were cultivated simultaneously to determine the protein concentration.

### 2.8. Carboxymethyl Lysine Determination

The PEE from *F. chiloensis* (62.5 *μ*g/mL) was added to a confluent culture of cells grown in 75 cm^2^ culture flasks. Cells were treated under both schemes of co- and preincubations with the PEE and challenged with AAPH 168 mM during 1 h. Then, the medium was removed by vacuum aspiration and cells were left to recover for 24 h with complete medium. At the end of the incubation, the medium was removed by vacuum aspiration and cells were scrapped-off in PBS. Cells were centrifuged at 3500 rpm for 10 min, and the pellet was resuspended in CelLytic. Protein concentration was determined by the BCA method [30]. Twenty *μ*g of each sample was boiled for 5 minutes in Laemmli sample buffer and loaded onto 12% (*w*/*v*) SDS-PAGE gels. Electrophoresis was performed at 100 V for 1-2 hours. Then, proteins were electrotransferred onto a Hybond nitrocellulose membrane (GE Healthcare) during 1 h at 100 V. The membrane was blocked with PBS containing 1% BSA and 0.1% Tween 20. Membranes were incubated with anti-CML primary antibody, at a dilution of 1/1000, and washed 3 times with PBS and then incubated with HRP-conjugated secondary antibody. Membranes were revealed with the ECL. Signal intensities were quantified with ImageJ software (NIH).

### 2.9. Statistical Analysis

Statistical differences between different treatments and their respective control were determined by one-way analysis of variance (ANOVA) followed by Dunnett's multiple comparison test. The level of significance was set at *P* < 0.05. All statistical analyses were carried out using the software SPSS 14.0 for Windows (IBM, Armonk, NY, USA).

## 3. Results

The ability of polyphenols from Chilean native berries to protect AGS cells against free radical-induced damage was assessed using two different approaches, namely, activation of intracellular antioxidant mechanisms and direct free radical scavenging. The former was assessed by preincubating AGS cells with PPEs during 16 hours and subsequently (after washing cycles) exposing the cells to a challenge by free radical source. In the case of the studies about the free radical scavenging and/or metal-chelating activity of PPEs, AGS cells were simultaneously incubated with each PEE and the free radical sources (coincubation). The protective effects were evaluated using two different sources of free radicals: AAPH and H_2_O_2_/CuSO_4_ (Fenton-type reaction).

**Figure 1:**
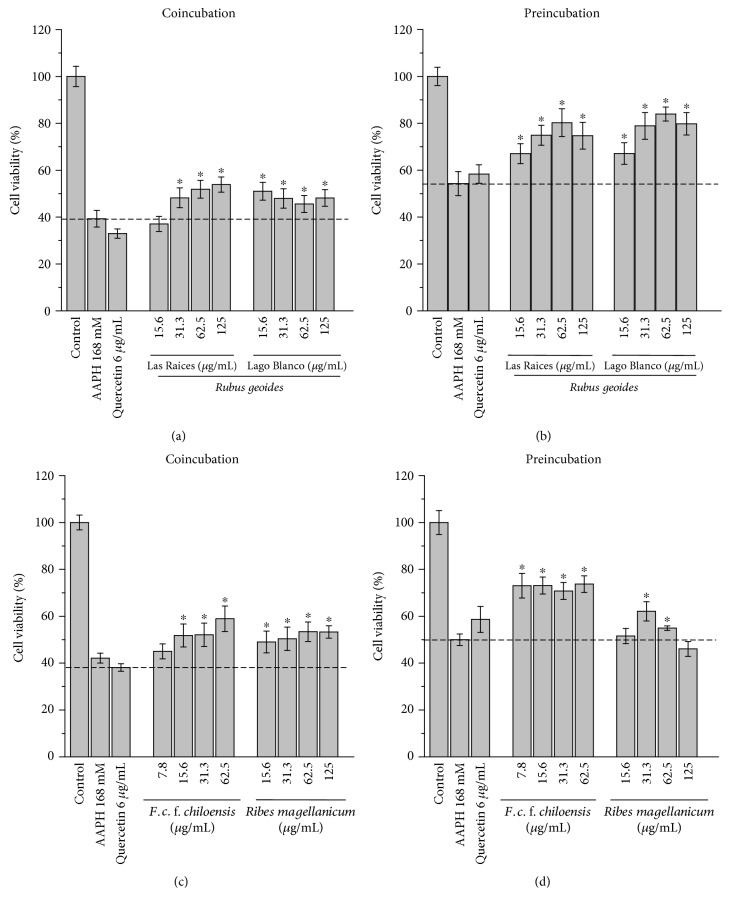
Effects of the PEEs on cell viability of AGS cells challenged with peroxyl radicals (AAPH, 168 mM). (a) Cytoprotective effects of the PEEs from *R. geoides* (Las Raices and Lago Blanco) in the coincubation model. (b) Cytoprotective effects of the PEEs from *R. geoides* (Las Raices and Lago Blanco) in the preincubation model. (c) Cytoprotective effects of the PEEs from *F. chiloensis* and *R. magellanicum* in the coincubation model. (d) Cytoprotective effects of the PEEs from *F. chiloensis* and *R. magellanicum* in the preincubation model.

In the absence of a free radical source, viability of AGS cells was not affected by pre- or coincubations with the different PPEs (15–125 *μ*g/mL) (data not shown). But, in the absence of PPEs, when AGS cells were exposed to different doses of free radicals generated by thermal decomposition of AAPH, cell viability showed an IC_50_ value of 168 mM.  Figure 1 shows the cellular viability for AGS cells challenged with AAPH (168 mM) and the protective effects of coincubations and preincubations with quercetin (positive control) or PEEs from the Chilean native berries *R. geoides*, *R. magellanicum*, and *F. chiloensis*. The addition of different concentrations of all the extracts (co- and preincubated) to AGS cell cultures induced a statistically significant cytoprotection (*P* < 0.05) against the challenge with AAPH-derived peroxyl radicals (Figures 1(a), 1(b), 1(c), and 1(d)). The Chilean strawberry *Fragaria chiloensis* was the most efficient species against free radical damage induced by AAPH, considering that its cytoprotective activity was exerted at lower concentrations of the PEEs (Figures 1(a) and 1(c)).

**Figure 2:**
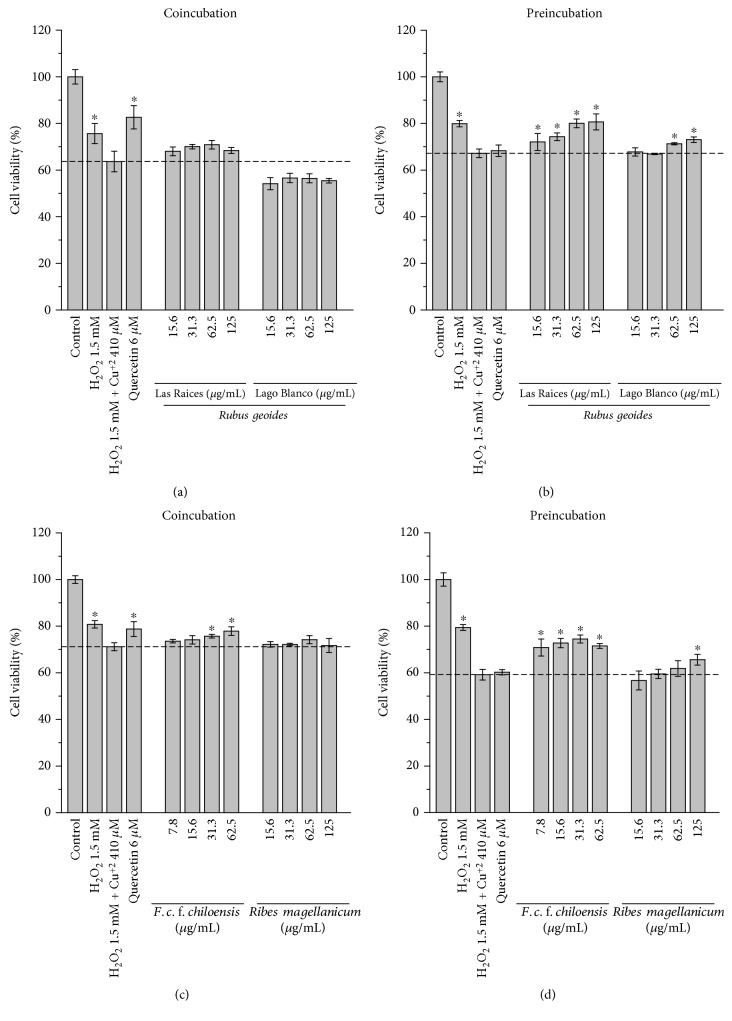
Effects of the PEEs in cell viability of AGS cells challenged with H_2_O_2_ (1.5 mM) + Cu^2+^ (410 *μ*M). (a) Cytoprotective effects of the PEEs from *Rubus geoides* (Las Raices and Lago Blanco) in the coincubation model. (b) Cytoprotective effects of the PEEs from *R. geoides* (Las Raices and Lago Blanco) in the preincubation model. (c) Cytoprotective effects of the PEEs from *Fragaria chiloensis* and *Ribes magellanicum* in the coincubation model. (d) Cytoprotective effects of the PEEs from *F. chiloensis* and *R. magellanicum* in the preincubation model.

**Figure 3:**
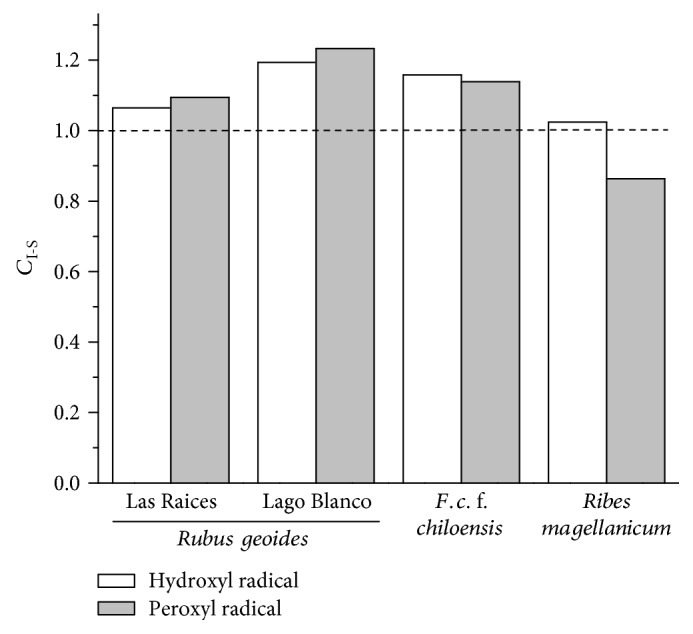
Ratio between normalized areas of cell viability plots for AGS cells preincubated with the PEEs over normalized area of AGS cells coincubated with PEEs and challenged with peroxyl radicals (AAPH, 168 mM) and hydroxyl radicals (H_2_O_2_, 1.5 mM + Cu^2+^, 410 *μ*M).

Figure S2 shows the effect of different concentrations of Cu^2+^ (added as CuSO_4_) in cell viability in the presence and absence of H_2_O_2_ (1.5 mM). It was found that, in the absence of H_2_O_2_, Cu^2+^ at 410 *μ*M did not affect the cell viability. However, in the presence of H_2_O_2_, cell viability decreased to 54.3 ± 6.6% (Figure S2). On the other hand, 1.5 mM H_2_O_2_ (in the absence of Cu^2+^) decreased cell viability by 14.3 ± 4% (data not shown).  Figure 2 shows that H_2_O_2_ + Cu^2+^ induce a significant decrease in the cell viability compared to the H_2_O_2_ controls (in the absence of Cu^2+^). Therefore, cytotoxic effects induced by H_2_O_2_ + Cu^2+^ can be attributed not only to free radicals but also in less degree to H_2_O_2_. Figures 2(a), 2(b), 2(c), and 2(d) show the protective effects of co- and preincubations with PEEs from *R. geoides*, *R. magellanicum*, and *F. chiloensis* against the damage induced by free radicals generated from the redox couple H_2_O_2_ + Cu^2+^. The positive control quercetin induced significant cytoprotection in the coincubation model, but not in the preincubation conditions (Figure 2). The protective effect of all the PEEs against the damage induced by H_2_O_2_ + Cu^2+^ was more efficient in the preincubation model than in the coincubation model. In the *F. chiloensis*, PEE presented a significant protection in the model of coincubation. To determine the prevalence of the cytoprotective mechanisms, the area under the curve was calculated using the cell viability plots against the different concentrations of the PEEs. The area under the curve was calculated by normalizing the ratio of the cell viability over cytotoxicity induced by the stressor agent. We defined the coefficient *C*_I-S_ (intracellular-scavenging) as the area under the curve of the protective effects induced by intracellular antioxidant mechanisms over the area under the curve of protective effects induced by extracellular scavenging. Therefore, when *C*_I-S_ = 1, cytoprotective effects of intracellular antioxidant mechanisms will be the same as that of direct scavenging; when *C*_I-S_ < 1, cytoprotective effects of scavenging will prevail over those exerted by intracellular activation; and when *C*_I-S_ > 1, cytoprotective effects of intracellular activation will prevail over those exerted by a direct scavenging of free radicals (or chelating effect on Cu^2+^).  Figure 3 shows the ratio *C*_I-S_ obtained by the analysis of the protection of the different PEEs against damage mediated by AAPH and H_2_O_2_ + Cu^2+^. It can be observed that for hydroxyl radicals the prevalent cytoprotective mechanism was the intracellular activation of antioxidant responses. This fact was also observed when peroxyl radicals were used as the stressor agent, except with the native currant *R. magellanicum*. In this case, the prevalent mechanism was the free radical scavenging.

**Figure 4:**
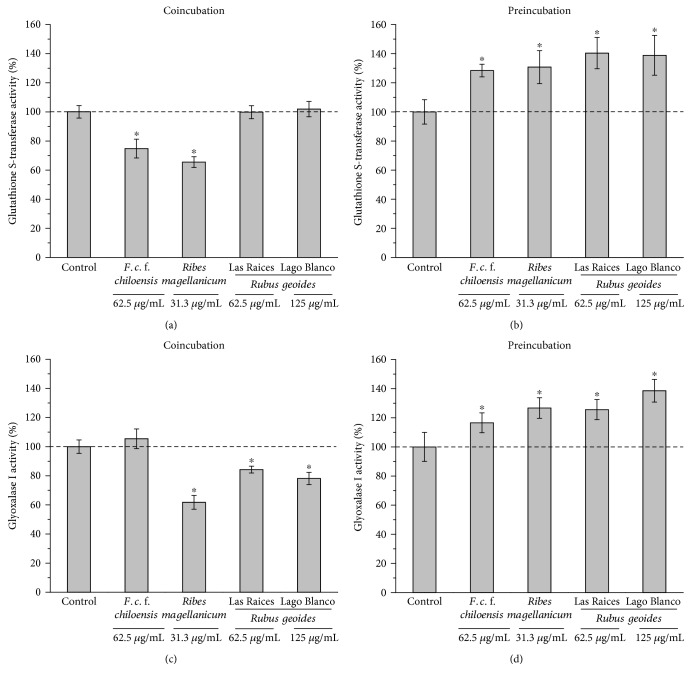
Effects of the PEEs in the enzymatic activity of glutathione S-transferases and glyoxalase I in AGS cells. (a) Glutathione S-transferase activity after coincubation of AGS with PEEs. (b) Glutathione S-transferase activity after preincubation of AGS cells with PEEs. (c) Glyoxalase I activity after coincubation of AGS with PEEs. (d) Glyoxalase I activity after preincubation of AGS with PEEs.

To verify the differences in both cytoprotective mechanisms induced by pre- and coincubations, the enzymatic activities of Nrf2-regulated enzymes were determined for both models with the PEEs without free radical challenge. Glutathione S-transferases and glyoxalase I activities were quantified after 30 minutes of incubation (coincubation model) or after 16 hours of incubation (preincubation model) in AGS cells. The concentrations were chosen considering (1) the most effective ones for the protection against peroxyl radicals and (2) those concentrations that presented statistically significant protection with both incubation models.  Figure 4(a) shows that *R. geoides* (Las Raices and Lago Blanco samples) did not present significant differences with the control, whereas *F. chiloensis* and *R. magellanicum* presented a decrease of 25.2% and 34.5%, respectively, in the activities of glutathione S-transferases after coincubation.  Figure 4(c) shows that coincubation with *R. magellanicum* and *R. geoides* (Las Raices and Lago Blanco) decreases significantly the activity of glyoxalase I by 38.2, 15.8, and 21.9%, respectively. Coincubation with *F. chiloensis* did not present significant differences when compared with the control. Figures 4(b) and 4(c) show that preincubation of AGS cells with all PEEs elicits a significant increase in the activity of the enzymes glutathione S-transferase and glyoxalase I compared with the control.

With the aim to determine the effectiveness of each PEEs in the free radical scavenging process, in vitro assays were performed by means of the ORAC methods, using fluorescein and pyrogallol red as the target molecules. ORAC fluorescein (ORAC-FL) provides information about the stoichiometry of the reaction between the free radicals produced by AAPH thermal decomposition, and ORAC red pyrogallol (ORAC-PGR) indicates the reactivity of a sample towards such free radicals.  Table 1 depicts that the order of the antioxidant capacity of all the PEEs was the same in both methods, however, showing higher differences in the PGR-based methodology. The efficiency in the free radical scavenging was *F. chiloensis > R. magellanicum > Rubus* (Lago Blanco sample) *> Rubus* (Las Raices sample) (Table 1)*. Fragaria chiloensis* was selected to perform further analyses considering its better efficiency to induce cytoprotection through intracellular antioxidant mechanisms and free radical scavenging.

**Figure 5:**
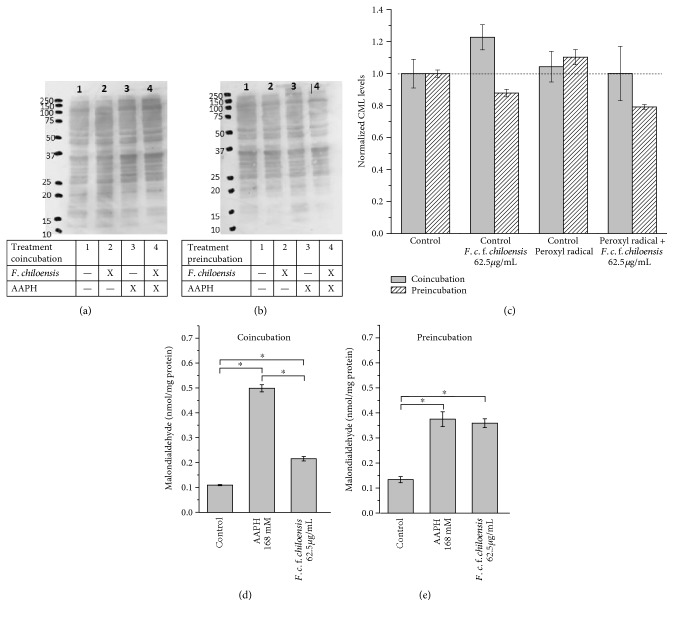
Effect of the PEE from *F. chiloensis* on oxidative stress markers. (a) and (b) Western blots showing the levels of carboxymethyl lysine (CML) in proteins from AGS cells co- and preincubated and challenged with peroxyl radicals (AAPH, 168 mM), respectively. (c) Densitometric analysis of CML levels obtained from the Western blots. (d) and (e) Thiobarbituric acid reactive substances expressed as malondialdeyde concentration (nmol/mg protein) in AGS cells co- and preincubated with the PEE from *F. chiloensis* and challenged with peroxyl radicals (AAPH, 168 mM).

The protective effects exerted by polyphenols from *F. chiloensis* in AGS cells in the co- and preincubation models challenged with AAPH (168 mM) were assessed using markers of oxidative stress such as the advanced glycation end product carboxymethyl lysine (CML) in proteins and thiobarbituric acid reactive substances (TBARs). Representative blots of CML under the different treatments described before are shown in Figure 5. The panel C displays a densitometric analysis of CML levels detected by immunochemical means (Figure 5). A slight increase of 4.3 ± 0.4% and 10.3 ± 0.2% in the CML levels was observed for samples incubated with peroxyl radicals for coincubation and preincubation, respectively, when compared to control experiments. On the other hand, we have only detected a significant decrease of CML levels in cells preincubated with the PEE from *F. chiloensis*. The levels of CML decrease in 20.9 ± 2.7% and 31.2 ± 4.9% when compared with the untreated and AAPH controls, respectively.

Figures 5(d) and 5(e) show the TBAR levels in AGS cells exposed to peroxyl radicals and submitted to co- and preincubation with the PEE from *F. chiloensis*.  Figure 5(d) presents an increase of 358.7% in the levels of MDA when AGS cells were coincubated with the free radicals only. On the other hand, when cells were coincubated with the PEE, the increase in MDA was only 95%. The inhibition of the MDA levels induced by the PEE in the model of coincubation was statistically significant when compared with the peroxyl radical control (*P* < 0.05).

In the presence of AAPH, when cells were treated employing the preincubation model, in comparison with control experiments, an increase of 178.8% in the MDA levels was observed (Figure 5(e)). When AGS cells were preincubated with the PEE and posteriorly challenged with AAPH-derived peroxyl radicals, an increment of 171.2% was observed (Figure 5(e)). Therefore, employing the preincubation approach, no significant decrease in the MDA levels was detected in the presence of PEEs.

## 4. Discussion

It has been established that one of the main sites of the biological action of polyphenols is the stomach [35]. For instance, polyphenols from coffee and red wine can exert their antioxidant effects at the stomach cavity during postprandial processes decreasing the levels of plasmatic malondialdehyde [36–38]. On the other hand, lipid peroxidation reactions produced during digestion are favored due to the acidic environment of the stomach [13]. This fact could increase the potential damage mediated by free radicals, which have been proposed to play a relevant role in numerous diseases including stomach ulcers and cancer [39]. Polyphenols can induce protective effects against free radical-induced damage by multiple mechanisms including metal-ion complexation, free radical scavenging, and activation of intracellular detoxifying mechanisms [40, 41]. However, the prevalence of the protective mechanisms induced by polyphenols in epithelial gastric human cells has been poorly understood.

In this work, we assessed the prevalence of protective mechanisms mediated by polyphenols against the damage induced by free radicals generated by the redox couple H_2_O_2_ + Cu^2+^ and thermal decomposition of AAPH, which produce hydroxyl [28] and peroxyl radicals [29], respectively.

Considering that free radical scavenging occurs typically in seconds (lifetime of peroxyl radicals in tissues has been estimated to be 7 s) [42] and the activation of intracellular protective mechanisms comprises several hours [43], we have hypothesized that coincubations of AGS cells with the PEEs and the free radical sources might reflect protective effects by a direct free radical scavenging and/or metal chelation reactions. On the other hand, preincubations (16 h) of cells with the PEEs and a subsequent challenge with free radicals would indicate protective effects mediated by intracellular antioxidant mechanisms.

Cell viability of AGS cells pre- and coincubated with the different PEEs and exposed to AAPH-derived peroxyl radicals indicates that both mechanisms can induce significant protective effects (Figure 1). This fact agrees with numerous reports as well as with the bimolecular rate constants for the reactions between peroxyl radicals and linoleic acid or peroxyl radicals and chlorogenic acid (1 · 10^2^ M^−1^ s^−1^ and 1.28 · 10^5^ M^−1^ s^−1^, resp.) [23]. This allows the possibility of competitive reactions between cell components, such as membrane lipids and the polyphenols. To determine the prevalence of the main protective mechanisms induced by the studied PEEs, we used the ratio between the area under the curve of cytoprotection mediated by intracellular antioxidant mechanisms over the direct free radical scavenging (evaluated from coincubation conditions) (Figure 3). The analysis of the obtained data indicated that intracellular antioxidant mechanism (ICM) was the prevalent protective mechanism induced by polyphenols from the Chilean raspberry *R. geoides* (Las Raices and Lago Blanco samples) and the native strawberry *F. chiloensis*, with 9.5, 23.9, and 13.9%, respectively (Figure 3). Interestingly, only the polyphenols from the currant *Ribes magellanicum* showed that the direct free radical scavenging was the main protective mechanism with a 13.7% over ICM (Figure 3).

Employing the mixture H_2_O_2_/Cu^2+^ instead of AAPH-derived peroxyl radicals, the study of the cytoprotective effects mediated by PEEs showed, for the extracts of all berries species, that ICM was the prevalent cytoprotective mechanism. It is noteworthy that only the polyphenol employed as control (quercetin) and the extract of *F. chiloensis* presented a significant protection of AGS cells against the oxidative stress induced by H_2_O_2_/Cu^2+^ in the coincubation model. This is probably due to Cu^2+^ chelation, considering that complexation of Cu^2+^ by quercetin has been reported [44]. This fact is in agreement with the kinetics of the reactions mediated by hydroxyl radicals, which typically present second-order rate constants of ~10^9^ M^−1^ s^−1^ (close to diffusion limit) [23], and thus, a protection elicited by scavenging of hydroxyl radicals by polyphenols could not be expected. This behavior validates our findings with peroxyl radicals and provides empirical evidence indicating that polyphenols from our berries as well as vegetables and other fruits could not induce an efficient protection by means of the scavenging of hydroxyl radical [45].

To test the hypothesis that co- and preincubations of AGS cells with polyphenols and free radicals involve different mechanisms of cellular protection, we determined the enzymatic activity of Nrf2-regulated enzymes, which constitute one of the final outcomes of the Nrf2 downstream [45]. The transcription factor Nrf2 coordinates the expression of detoxifying enzymes for survival and defense in stressful conditions [45, 46]. For this purpose, the enzymatic activities of the detoxifying enzymes glyoxalase I (GLOI) and glutathione S-transferases (GST) were assessed. Co- and preincubations were performed with the concentrations of the PEEs that presented the best cytoprotective effects against peroxyl radicals. We observed that only preincubations with the PEEs induced a significant increase in the activity of both enzymes (Figure 4), whereas coincubations did not induce significant changes and even a decrease in the enzymatic activities was observed with some species (Figures 4(a) and 4(c)). A lag period of time between the initiation of the oxidative stress and the expression of Nrf2-regulated enzymes has also been reported in AGS cells stressed with H_2_O_2_, which have shown to increase the expression of the enzyme heme oxygenase-1 at least after 3 h poststress [43]. A time-dependent Nrf2-regulated response in the expression of glyoxalase I (with a maximum at 18 h after treatment) has also been reported for HepG2 hepatocytes incubated with the microbial compound monascin [47]. We have previously reported that preincubations (24 h) of AGS cells with the PEEs from *Rubus geoides* induce an increase in the levels of intracellular glutathione as well as protective effects against hydrogen peroxide and methylglyoxal [9].

The efficiency of the PEEs on the free radical scavenging mechanism was evaluated employing ORAC methods, using as target molecules fluorescein (ORAC-FL) and red pyrogallol (ORAC-PGR). ORAC-FL has been related to the stoichiometry of the reaction between AAPH-derived peroxyl radicals and the antioxidant compound, while ORAC-PGR has been associated with the reactivity (rate of reaction) of polyphenols towards such free radicals [33]. The PEE from *F. chiloensis* presented the highest value of ORAC-FL and ORAC-PGR, indicating that the PEE from this species was the most efficient to inactivate peroxyl radicals in terms of the stoichiometry of the reaction as well as in terms of the reactivity of its polyphenolic compounds. The phenolic composition of the PEEs from *R. geoides*, *Ribes* spp., and *F. chiloensis* has been previously reported. The PEEs from the selected berries present antioxidant activity, but the chemical composition of the fruit phenolics is very different, as can be expected from different fruit (berries) species [9, 16, 24–26]. The PEE of *Fragaria chiloensis* presents hydrolizable and condensed tannins as well as flavonoids, while *Ribes* spp. are rich in anthocyanins, caffeoylquinic acids, and flavonoids and *Rubus geoides* yielded flavonoid glycosides and tannins [9, 16, 24–26].

Considering that the PEE from *F. chiloensis* was the most efficient sample to scavenge peroxyl radicals and also presented a high protection mediated by ICM, we selected this species to carry out additional experiments. We evaluated the cytoprotective effect of the PEE from *F. chiloensis* using two different oxidative stress markers, namely, carboxymethyl lysine (CML) and TBAR levels in both experimental models. Carboxymethyl lysine results from the nonenzymatic posttranslational modification occurring in proteins and is one of the most abundant advanced glycation end products in the human body [48]. Carboxymethyl lysine levels have shown to be increased in aged tissues, constituting a marker of occurrence of glycoxidative reactions during aging [49–52]. This compound can be generated intracellularly by direct reaction with glyoxal, which can be detoxified intracellularly by the glyoxalase system (comprised by glyoxalase I and glyoxalase II) [53]. Consequently, a decrease in glyoxalase I activity has been associated with increased CML levels [54]. We have found that the PEE from *F. chiloensis*, in the preincubation model, was able to protect AGS cells from the glycoxidative damage reducing the CML relative levels, compared with the peroxyl radical control (Figures 5(a), 5(b), and 5(c)). This result agrees with the increased levels of electrophile-detoxifying enzymes such as GLOI and GST after preincubation with the PEE from *F. chiloensis* (Figure 4). This constitutes an interesting finding, considering that the overexpression of GLOI has been shown to increase the longevity of the nematode *C. elegans* [54] and to retard the senescence of renal proximal tube epithelial cells [55].

The effect of peroxidative damage to lipids was determined using the TBAR method (Figures 5(d) and 5(e)). Only the coincubation model presented a significant decrease of malondialdehyde levels, implying an inhibition of lipid peroxidation process. It has been reported that Nrf2 activation mediated by lucidine in human keratinocytes (HaCaT) cells can induce a decrease in MDA levels induced by the presence of AAPH [56]. The differences observed in the work of Kumar et al. [56] and our results are probably due to different experimental designs. Such authors treated the HaCaT cells with AAPH during 6 h, a time that should be enough for MDA diffusion from the cell membrane and to be detoxified intracellularly. We incubated with AAPH during 1 h and immediately analyzed the levels of MDA. Nonetheless, our results indicate that despite the effectiveness of coincubation in reducing the MDA levels due to the fast kinetics of free radical scavenging mechanisms, the most relevant cytoprotective mechanism induced by the PEEs from the selected native berries was achieved by intracellular antioxidant mechanisms.

## 5. Conclusions

Our results with the Chilean berries show the potential of these native fruits as sources of natural antioxidants exerting protective effects on AGS cells against free radical-induced damage. Such effect is related to a direct scavenging activity of polyphenols towards free radicals and also to the modulation of pivotal intracellular mechanisms. However, our results clearly demonstrate that the intracellular antioxidant response activation is the main mechanism accounting the protective effect of Chilean berries. To the best of our knowledge, this work is the first attempt for developing a model to study the prevalence of cytoprotective mechanisms mediated by polyphenols against the damage induced by free radicals in cell cultures. Establishing the differences in the prevalence of protective mechanisms induced by polyphenols may be useful in the design of novel strategies to maximize the efficiency of healthy effects mediated by dietary polyphenols.

## Supplementary Material

Figure S1. Chilean native berries used in this study: Rubus geoides (A), Ribes magellanicum (B) and Fragaria chiloensis ssp. chiloensis f. chiloensis (white fruits) (C). Figure S2. Effect of the Fenton type reaction on cell viability in AGS cells. The black line represents the cell viability of AGS cells exposed to different concentrations of Cu+2. The red line depicts the cell viability of AGS cells exposed to different concentrations of Cu+2 in the presence of H2O2 (1.5 mM).

## Figures and Tables

**Table 1:** Oxygen radical absorbance capacity (ORAC) of polyphenolic-enriched extracts from the selected native species using as free radical target fluorescein (FL) and red pirogallol (PGR).

Sample	ORAC-FL (mmol TE^a^/L)	ORAC-PGR (mmol TE^a^/L)
*Rubus geoides* (Las Raices)	4.26 ± 0.2	1.99 ± 0.4
*Rubus geoides* (Lago Blanco)	4.36 ± 0.4	3.55 ± 0.3
*Fragaria chiloensis*	7.16 ± 0.1	10.56 ± 1.1
*Ribes magellanicum*	5.11 ± 0.7	7.19 ± 0.1

^a^Trolox equivalents.

## Abbreviations

AAPH: 2,2′-Azobis(2-methyl-propionamidine) dihydrochloride
AGS cells: Human gastric adenocarcinoma cell line
CML: Carboxymethyl lysine
GLOI: Glyoxalase I
GST: Glutathione S-transferases
ICM: Intracellular antioxidant mechanisms
Nrf2: Nuclear factor-erythroid 2 p45
ORAC: Oxygen radical absorbance capacity
PEEs: Polyphenolic-enriched extracts
TBARs: Thiobarbituric acid reactive species.
