# Supplementary material for: Cytoprotective Mechanisms Mediated by Polyphenols from Chilean Native Berries against Free Radical-Induced Damage on AGS Cells
Source: Oxid Med Cell Longev. 2017 May 2;2017:9808520. doi: 10.1155/2017/9808520 (PMC5434242; doi:10.1155/2017/9808520)
Supplement: Supplementary file 1 — Figure S1. Chilean native berries used in this study: Rubus geoides (A), Ribes magellanicum (B) and Fragaria chiloensis ssp. chiloensis f. chiloensis (white fruits) (C). Figure S2. Effect of the Fenton type reaction on cell viability in AGS cells. The black line represents the cell viability of AGS cells exposed to different concentrations of Cu+2. The red line depicts the cell viability of AGS cells exposed to different concentrations of Cu+2 in the presence of H2O2 (1.5 mM). [file 9808520.f1.docx]

Supplementary material

| 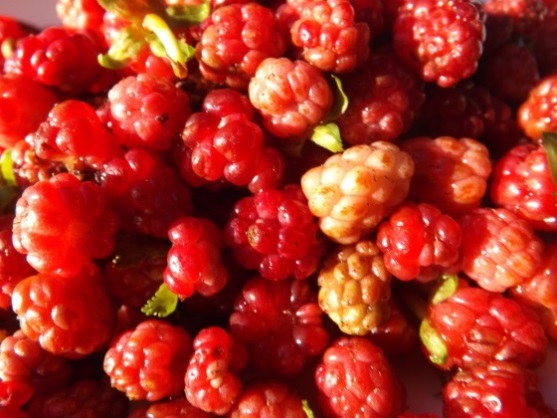  A |
| --- |
| 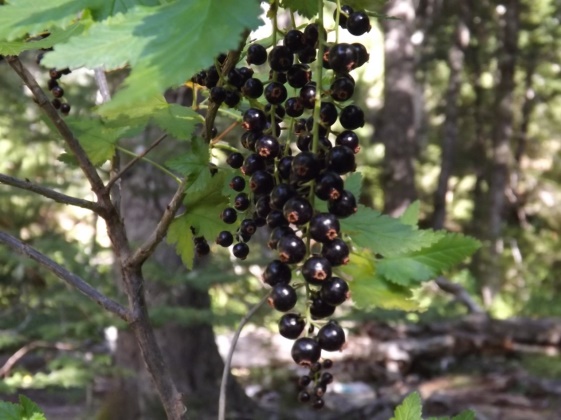  B |
| 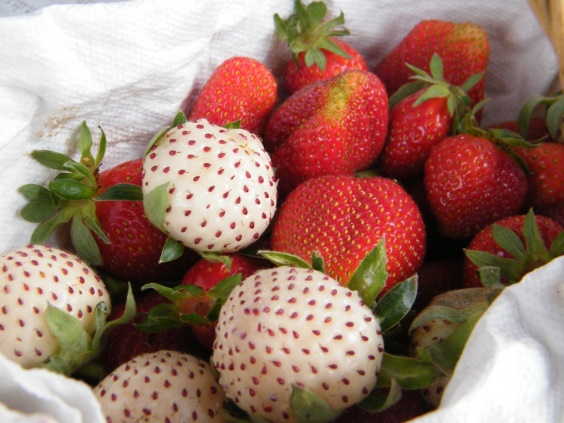  C |

Fig. S1. Chilean native berries used in this study: *Rubus geoides* (A), *Ribes magellanicum* (B) and *Fragaria chiloensis* ssp. *chiloensis* f. *chiloensis* (white fruits) (C).

Fig. S2. Effect of the Fenton type reaction on cell viability in AGS cells. The black line represents the cell viability of AGS cells exposed to different concentrations of Cu^+2^. The red line depicts the cell viability of AGS cells exposed to different concentrations of Cu^+2^ in the presence of H_2_O_2_ (1.5 mM).
